# Aphid facultative symbionts reduce survival of the predatory lady beetle *Hippodamia convergens*

**DOI:** 10.1186/1472-6785-14-5

**Published:** 2014-02-20

**Authors:** Kelly Costopoulos, Jennifer L Kovacs, Alexandra Kamins, Nicole M Gerardo

**Affiliations:** 1 Department of Biology, O. Wayne Rollins Research Center, Emory University, 1510 Clifton Road N.E, Atlanta, GA 30322, USA; 2 Department of Biology, Spelman College, 350 Spelman Lane, S.W, Atlanta, GA 30314, USA

**Keywords:** Symbiosis, Mutualism, Symbiont-conferred protection, *Serratia symbiotica*, *Hamiltonella defensa*, Pea aphid, *Acyrthosiphon pisum*, Predation, Defensive mutualism, Protective symbiosis

## Abstract

**Background:**

Non-essential facultative endosymbionts can provide their hosts with protection from parasites, pathogens, and predators. For example, two facultative bacterial symbionts of the pea aphid (*Acyrthosiphon pisum*), *Serratia symbiotica* and *Hamiltonella defensa,* protect their hosts from parasitism by two species of parasitoid wasp. Previous studies have not explored whether facultative symbionts also play a defensive role against predation in this system. We tested whether feeding on aphids harboring different facultative symbionts affected the fitness of an aphid predator, the lady beetle *Hippodamia convergens*.

**Results:**

While these aphid faculative symbionts did not deter lady beetle feeding, they did decrease survival of lady beetle larvae. Lady beetle larvae fed a diet of aphids with facultative symbionts had significantly reduced survival from egg hatching to pupation and therefore had reduced survival to adult emergence. Additionally, lady beetle adults fed aphids with facultative symbionts were significantly heavier than those fed facultative symbiont-free aphids, though development time was not significantly different.

**Conclusions:**

Aphids reproduce clonally and are often found in large groups. Thus, aphid symbionts, by reducing the fitness of the aphid predator *H. convergens*, may indirectly defend their hosts’ clonal descendants against predation. These findings highlight the often far-reaching effects that symbionts can have in ecological systems.

## Background

Close symbiotic associations between microbes and invertebrates are nearly ubiquitous, particularly among insects [1-4]. Though some microbial symbionts are parasitic and have detrimental fitness effects for their hosts, many symbionts provide their hosts with fitness benefits [5-7]. These mutualistic symbionts are generally vertically transmitted from parent to offspring and can be either essential to host survival (obligate, primary) or facultative (secondary) [8,9]. Primary symbionts often provide essential nutrients to their hosts, as in the case of *Buchnera aphidicola*, an obligate bacterial symbiont of most aphid species [10]. While facultative symbionts are not necessary for survival, they can provide diverse benefits to their hosts, including protection from pathogenic microbes, parasites, and predators [11-14].

Symbionts have been found to provide hosts with protection against pathogenic micro-organisms in a wide range of invertebrate systems [6,11,15]. For example, *Wolbachia* bacteria, well-known for killing infected male larvae, can also protect *Drosophila* flies against RNA viruses [16]. Several other microbial symbionts produce antifungal substances that protect their crustacean hosts against the pathogenic fungus *Lagenidium callinectes*[14,17]. Similarly in aphids, the facultative symbiont *Regiella insecticola* increases host resistance against several fungal pathogens [18,19].

Additionally, some symbionts protect their host from parasitic invertebrates, including parasitic wasps and helminthes [6,20-22]. Both *Serratia symbiotica* and *Hamiltonella defensa,* two facultative symbionts of the pea aphid *Acyrthosiphon pisum*, protect their host against parasitoid wasps [13]. In the case of *H. defensa*, this protection is in part due to a toxin produced by an associated bacteriophage [23]. While the presence of the symbiont and bacteriophage do not affect wasp attack rates or initiation of wasp embryogenesis and larval development, approximately five days after wasp oviposition, aphids with *H. defensa* are significantly more likely to contain dead wasp larvae than those without the facultative symbiont [23].

Similarly, a few symbionts are known to protect against predation. Most known cases of symbiont-conferred predator protection involve the production of toxic compounds by the symbiont. *Paederus* beetles, for example, harbor toxin-producing symbionts that deter predation by wolf spiders [24-26]. In marine environments, it is believed that chemically mediated defenses are often of symbiont origin [22]. For example, toxin-producing symbionts make larvae of the bryozoan *Bugula neritina* less palatable to fish predators [27].

Here, we determine whether two facultative symbionts of the pea aphid, *H. defensa* and *S. symbiotica*, protect their hosts against a major aphid predator, the lady beetle *Hippodamia convergens,* by altering predator fitness or feeding rate. We measure several components of fitness for beetles fed diets of aphids both with and without facultative symbionts. Based on the negative impact of these symbionts on parasitic wasp development [23], we predicted that facultative symbionts may also play a role in defense against predation. Specifically, we predicted that the presence of facultative symbionts in the diet of the predatory lady beetle larvae could decrease beetle survival, thereby lowering the overall risk of predation for a population of aphids. This, in turn, could select for lower feeding rates on aphids with facultative symbionts. Though we did not detect any differences in lady beetle feeding rate on aphids with or without facultative symbionts, we did find that lady beetles fed aphids harboring facultative symbionts had significantly lower survival, and interestingly, significantly increased adult weight upon emergence.

## Methods

Adult lady beetles (*H. convergens*) were obtained from Carolina Biological Supply. Lady beetles were kept in mixed sex groups of approximately 20 and maintained at 25°C with a light regime of 16:8 Light:Dark. Adult beetles were fed aphids from genetically identical asexual aphid lineages harboring either the facultative symbiont *S. symbiotica* (aphid line 5AR), the facultative symbiont *H. defensa* (aphid line 5AT), or no facultative symbiont (aphid line 5AO). In this clonal aphid genotype, infection status and infection type do not significantly affect size or growth rate [28]. Aphids were reared on fava seedlings (*Vicia faba* L*.*) at 20°C with a light regime of 18:6 Light:Dark. New aphid bearing plants were supplied to the adult lady beetles daily.

Lady beetle egg clutches were removed from the adult cages daily and placed in individual Petri dishes. Once eggs hatched, larvae were separated into individual Petri dishes. Larvae were fed aphids from the same aphid line containing the same facultative symbiont that their parents ate. Larvae were fed fresh aphids *ad libitum*, and moist cotton balls were supplied and replaced as needed. Survival, time to pupation, and time to emergence from pupation of all larvae and pupae were recorded daily. Within 24 hours of emergence and prior to additional feeding, adult lady beetles were weighed to compare sizes and sexed using dimorphic features of the distal margin of the final abdominal sternite [29]. Weights at emergence were standardized to have a mean of zero and a standard deviation of one for males and females separately.

To determine the effect of aphid facultative symbionts on lady beetle larval survival from hatching, three replicates of the experiment were performed, hereafter referred to as “trials”. In two experimental trials (trials 1 & 2), lady beetle larvae were fed either aphids harboring *H. defensa* (5AT), *S. symbiotica* (5AR), or no facultative symbiont (5AO). These were the same aphids their parents had been fed. In trial 3, we were unable to collect data for larvae fed 5AR aphids, and so data was collected only for 5AT and 5AO aphids. In total, 345 lady beetle larvae were observed across the three trials; 148 were fed 5AT aphids, 73 were fed 5AR aphids, and 124 were fed 5AO aphids (Trial 1: *N* (5AR) = 54, *N* (5AT) = 54, *N* (5AO) = 50; Trial 2: *N* (5AR) = 19, *N* (5AT) = 48, *N* (5AO) = 38; Trial 3: *N* (5AT) = 26, *N* (5AO) = 56;).

In our statistical analyses of the effect of aphid symbiont on lady beetle survival, development time, and weight at emergence, trial is included as a cofactor. Right censored proportional hazard fit models with aphid symbiont type and trial as cofactors were performed in JMP® Pro 10.0 [30] after testing the assumption of proportional hazards in R 2.14.2 using cox.zph of the Survival package. Once individuals reach pupation, however, it is difficult to determine whether they are alive or not, and therefore pupa were determined to have died when they did not emerge as adults after two weeks. Thus, a finer scale survival analysis was performed as above on data from hatching to pupation, and survival from pupation to adult emergence was analyzed using a generalized linear model with a logit linked binomial distribution run on a subset of 107 individuals that pupated. Due to the small number of individuals fed aphids with facultative symbionts surviving to pupation, both pupae fed 5AT aphids and 5AR aphids were pooled into a “with symbiont” group for this analysis. Standardized least squares models were used to determine the effect of aphid facultative symbionts on lady beetle development time, both from hatching to pupation and from pupation to adult emergence. To determine the effect of the presence or absence of aphid facultative symbionts on adult lady beetle weight at emergence standard least squares models with aphid symbiont type and trial as cofactors were used for male and female beetles separately; larvae fed 5AT and those fed 5AR aphids were pooled for this analysis to provide a larger sample size of adult lady beetles that had been fed aphids with facultative symbionts as larvae. Additionally, we tested whether the sex ratio of emerging adults was significantly different from the expected 0.50 probability for each sex using a two-sided chi-square test.

To explore the effect of aphid facultative symbiont on predator feeding rates, we recorded aphids consumed per day by paired adults (1 male and 1 female) for seven days. Lady beetle pairs were fed either 5AR, 5AT or 5AO aphids. During two trials in which adult lady beetle pairs were fed 5AT and 5AO aphids, forty unique pairs of lady beetles were placed on individual fava bean plants with 25 fourth instar aphids (Trial 1 *N* (5AO) = 15, *N* (5AT) = 8; Trial 2 *N* (5AO) = 10, *N* (5AT) = 7). In a third trial, another sixteen adult lady beetle pairs were fed either 5AR (*N* = 8) or 5AO (*N* = 8) aphids. In this trial, lady beetle pairs were placed on individual fava bean plants with 30 fourth instar aphids. Each day for seven consecutive days, the remaining aphids were counted and lady beetle pairs were transferred to a new plant with 25 or 30 aphids, depending on the trial. To determine whether adult lady beetles fed aphids with the facultative symbionts consumed more or less aphids than those fed symbiont-free aphids, we used ANOVAs to determine whether the total number of aphids eaten by the pairs over the seven day period differed significantly between those fed aphids that were symbiont positive (5AT or 5AR) or negative (5A0).

## Results

Aphid facultative symbionts significantly lower lady beetle larvae survival from hatching to adulthood, but do not affect pupal survival. Larvae fed 5AR aphids were 1.76 times more likely to die before emerging as adults than those fed symbiont free 5AO aphids (*p* = 0.0008), and larvae fed 5AT aphids were 1.68 times more likely to die before emerging as adults than those fed 5AO aphids (*p* = 0.005). There was no significant difference in survival from egg hatching to adulthood between those larvae fed 5AT aphids and those fed 5AR (*p* = 0.78). Overall mortality was significantly higher in trial 1, while there was no significant difference in mortality between trials 2 and 3. (Right censored proportional hazards model, survival from hatching to adult emergence = type of aphid eaten + trial + type of aphid eaten*trial, whole model: $\chi_{7}^{2}\;=122.42$, *p* < 0.0001, trial: $\chi_{2}^{2}\;=66.44$, *p* < 0.0001; type of aphid eaten: $\chi_{2}^{2}\;=19.02$, *p* = 0.0002, aphid eaten*trial: $\chi_{1}^{2}=16.99$, *p* = 0.0007).

**Figure 1:**
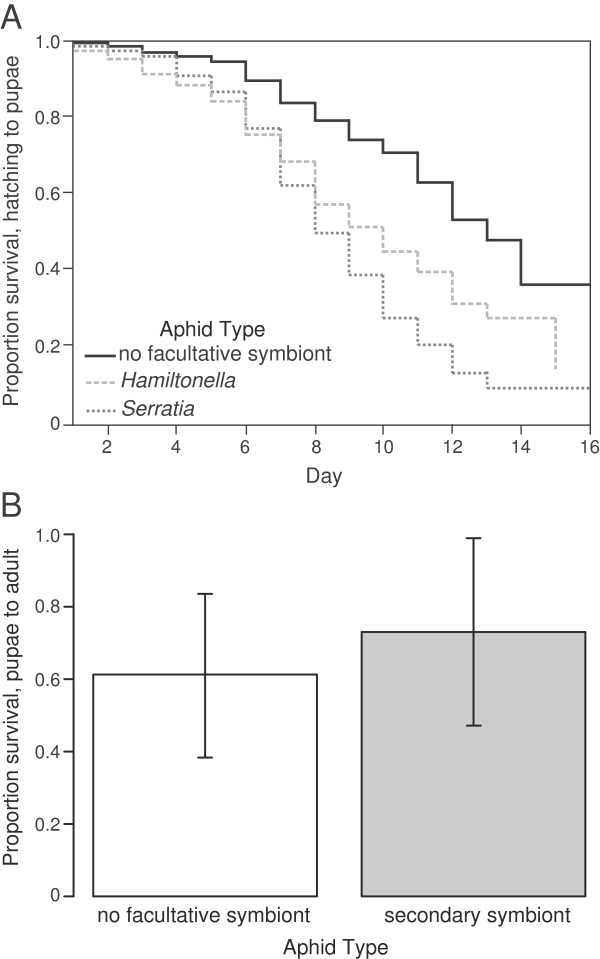
**Survival effects of aphid facultative symbionts on their lady beetle predators. A)** Aphid facultative symbionts significantly reduce lady beetle larval survival from hatching to pupation. **B)** Aphid facultative symbionts, however, do not significantly reduce survival between pupation and adult emergence. Error bars are +/− s.e.m.

At a finer scale, we assessed survival during each of the two major developmental stages, from hatching to pupation and from pupation to adulthood. Lady beetle larvae fed 5AR aphids (with *S. symbiotica*) were 2.56 times more likely to die before pupation than those fed 5AO aphids (with no facultative symbiont) (*p* < 0.0127, Figure 1A). Larvae fed 5AT aphids (with *H. defensa)* were 2.60 times more likely to die before pupation than those fed 5AO aphids (*p* < 0.0001, Figure 1A). There was no significant difference between larvae fed 5AR and those fed 5AT aphids in survival to pupation (*p* = 0.96, Figure 1A). While there was a significant effect of trial, with overall mortality being significantly higher in trial 1 than in the other two trials (no significant difference in survival between trials 2 and 3), larvae fed symbiont-containing aphids had lower survival across all three trials (Right censored proportional hazards model, survival to pupation = trial + type of aphid eaten + trial* type of aphid eaten, whole model: $\chi_{7}^{2}=108.35$, *p* < 0.0001; trial: $\chi_{2}^{2}=56.35$, *p* < 0.0001; type of aphid eaten: $\chi_{2}^{2}\;=22.91$, *p* < 0.0001, trial* type of aphid eaten = $\chi_{3}^{2}\;=12.96$, *p* = 0.005, Figure 1A). Unlike larval survival from hatching to pupation, there was no significant effect of aphid facultative symbiont presence on the survival of lady beetle pupae to adult emergence (Figure 1B). Again, overall mortality was significantly higher in trial 1, but there was no significant interaction between trial and the presence of aphid symbionts in the lady beetle diet (Generalized linear model, binomial distribution, logit linked, survival to adult emergence = trial + fed aphids with or without symbionts + trial *fed aphids with or without symbionts, whole model: $\chi_{3}^{2}\;=13.6$, *p* = 0.0035, trial: $\chi_{1}^{2}\;=13.05$, *p* = 0.0003, fed aphids with or without symbionts: $\chi_{1}^{2}\;=0.72$, *p* = 0.40, trial*fed aphids with or without symbionts: $\chi_{1}^{2}\;=1.72$, *p* = 0.19, Figure 1B).

Aphid facultative symbionts do not affect the time lady beetles spend in the larval or pupal stages. The type of aphid eaten did not affect the length of time an individual spent as a larva prior to pupation (Standard least squares model: time to pupation = trial + type of aphid eaten + type of aphid*trial, whole model: F_7,113_ = 1.69, *p* = 0.12; trial: d.f. = 2, F = 0.11, *p* = 0.74, type of aphid eaten: d.f. = 2,1.85, *p* = 0.18, type of aphid* trial: d.f. = 2, F = 2.03, *p* = 0.11; Average days from hatching to pupation, $\bar{x}\pm \mathrm{std}\;\mathrm{error}$, 5AO: 11.68 ± 0.13, 5AT: 11.74 ± 0.16, 5AR: 11.74 ± 0.38). Due to the high overall mortality observed in trial 1, the effect of the presence or absence of aphid facultative symbionts on development time from pupation to adult emergence was only analyzed for trials 2 and 3. There was no significant effect of facultative symbiont presence in aphids on the time between pupation and emergence as an adult, though there was a significant difference between trials in the length of pupation (Standard least squares model: time in pupation = trial + fed aphids with or without symbnts + trial * fed aphids with or without symbionts , whole model: F_3,71_ = 4.19, *p* = 0.0087, trial: d.f. = 1, F = 10.26, *p* = 0.0020, fed aphids with or without symbionts: d.f. = 1, F = 1.93, *p* = 0.17, trial* fed aphids with or without symbionts: d.f. = 1, F = 1.22, *p* = 0.27; Average days in pupation, $\bar{x}\pm \mathrm{std}\;\mathrm{error}$, 5AO: 4.69 ± 0.13, 5AT & 5AR: 4.87 ± 0.86).

**Figure 2:**
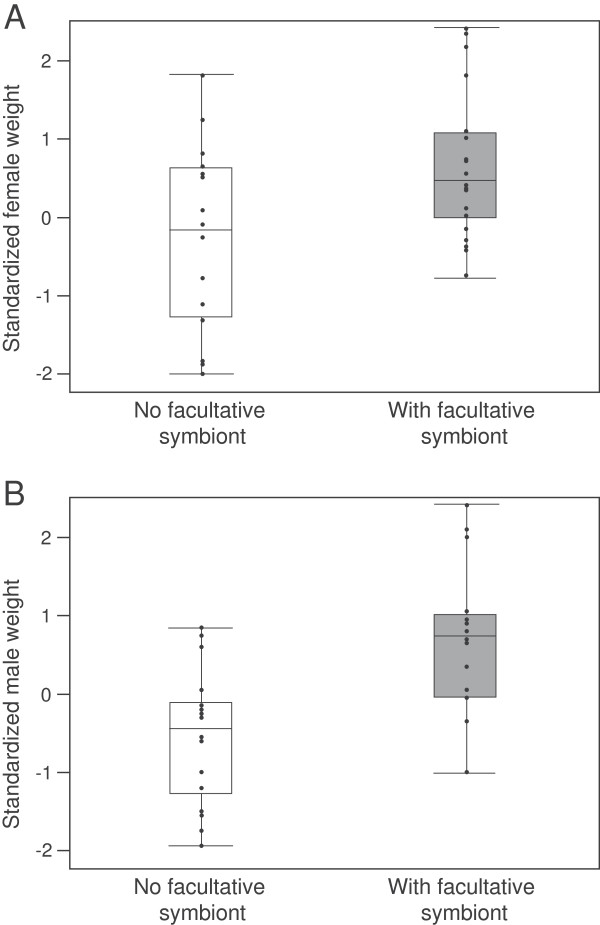
**Surviving lady beetles fed aphids with facultative symbionts during development have significantly increased final weight. A)** Female lady beetles fed aphids with no symbiont or fed aphids with a facultative symbiont (trials with aphid with *S. symbiotica* or with *H. defensa* combined). **B)** Male lady beetles fed aphids with no symbiont or fed aphids with a facultative symbiont.

Aphid facultative symbionts do not affect sex-specific survival, but do have a significant effect on lady beetle weight at emergence from pupation. The presence or absence of facultative symbionts in the aphid diet of lady beetle larvae did not alter the 50/50 sex ratio of emerging adults (Likelihood ratio: *χ*^2^  = 0.22, p = 0.64). Therefore there was no sex-specific effect of aphid facultative symbiont on lady beetle larvae survival to adulthood. However, we did find a significant effect of lady beetle larval diet (facultative symbiont-free or with facultative symbionts) on wet mass at emergence, and trial did not affect male or female lady beetle weight at emergence (Standard least squares model, Males: weight at emergence = trial + fed aphids with or without facultative symbionts, whole model: F_3,30_ = 6.35, *p* = 0.002; trial: d.f. = 2, F = 0.84, *p* = 0.44, fed aphids with or without symbionts: d.f. = 1, F = 18.6, *p* = 0.0002; Females: weight at emergence = trial + fed aphids with or without facultative symbionts, whole model: F_34_ = 4.68, *p* = 0.008; trial: d.f. = 2, F = 2.94, *p* = 0.06, fed aphids with or without symbionts: =0.95, *p* = 0.002, Figure 2). Overall, male adult lady beetles weighed 14.1 ± 2.1 mg, and female lady beetles weighed 17.4 ± 3.4 mg at emergence. Both males and females that were fed aphids with facultative symbionts were significantly heavier at the time of emergence than lady beetles that had been fed aphids without facultative symbionts (ANOVA, Males: F_1,32_ = 17.56, *p* = 0.0002; Females: F_1,36_ = 7.36, *p* = 0.01, Figure 2). Females fed aphids without symbionts weighed 15.4 ± 3.3 mg at emergence, while those fed aphids with symbionts were significantly heavier (5AR fed females: 21.6 ± 3.4 mg at emergence; 5AT fed females: 18.6 ± 2.6 mg at emergence). The same pattern was seen in male weight at emergence. Males fed aphids without symbionts weighed 12.9 ± 1.7 mg at emergence, males fed 5AT aphids weighed an averaged of 15.4 ± 1.9 mg at emergence, and the one male fed 5AR weighed 15.6 mg at emergence.

**Figure 3:**
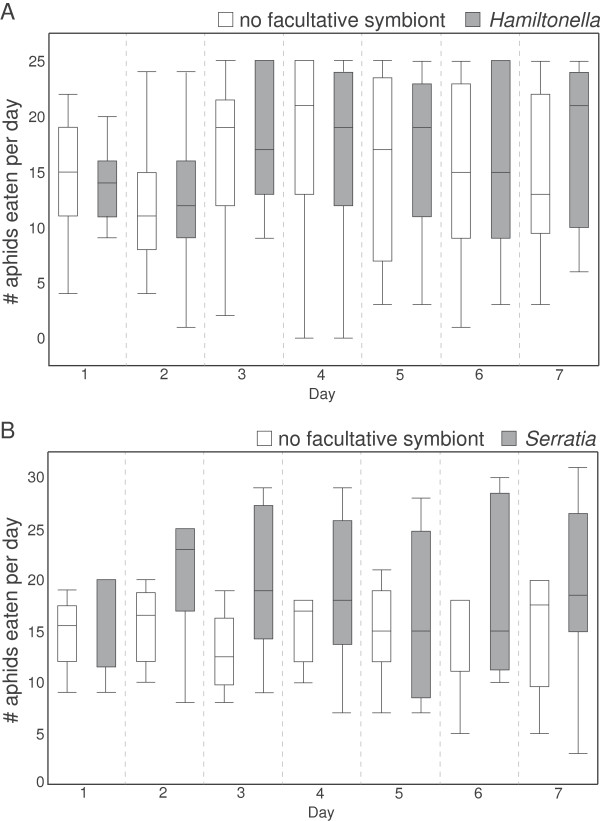
**Aphid facultative symbionts do not significantly impact adult lady beetle feeding rate. A)** Combined results of two trials in which adult lady beetle pairs were fed aphids either with or without the *Hamiltonella* symbiont (5AT = with *Hamiltonella*, 5AO = without *Hamiltonella*). **B)** Results of a third trial in which adult lady beetle pairs were fed aphids either with or without the *Serratia* symbiont (5AR = with *Serratia*, 5AO = without *Serratia*).

Aphid facultative symbionts do not affect lady beetle feeding rates. In the three 7-day feeding trials run, we saw no significant effect of aphid facultative symbiont presence or type on lady beetle feeding rates (ANOVAs; Trial 1 5AT & 5A0, d.f. = 1, F = 0.43, *p* = 0.52, Trial 2 5AT & 5A0, d.f. = 1, F = 1.85, *p* = 0.19; Trial 3 5AR & 5A0, d.f. = 1, F = 0.64, *p* = 0.44; Figure 3A & B). Therefore, lady beetle predation rates are not affected by the presence or type of facultative symbionts in their aphid prey.

## Discussion

We found there was a significant effect of aphid symbionts on lady beetle survival from egg hatching to pupation and weight at adult emergence. The presence of either of the two facultative symbionts in the aphid prey of lady beetles did not affect how long it took larvae to reach pupation or for pupae to emerge as adults. There was no effect of larval diet on sex-specific survival (adult sex ratio), nor was there any effect of facultative symbiont on lady beetle feeding rate. Despite their similar impacts on predator survival and weight, the mechanisms by which the two symbionts, *H. defensa* and *S. symbiotica*, affect lady beetle survival may be entirely different.

Several bacterial symbionts are known to affect predator–prey relationships. For example, *Paederus* beetle larvae are protected from wolf spider predation by a polyketide toxin pederin, which is produced by its bacterial symbiont [26,31,32]. Symbiotic plant fungal endophytes can also affect plant herbivores. For example, the fungus *Neotyphodium lolil* deters insect herbivory of the perennial ryegrass by producing an alkaloid, peramine [32]. Interestingly, lady beetles fed on aphids reared on *Neotyphodium lolii* infected grasses exhibit reduced fecundity, extended larval development, and reduced survival [33]. These results demonstrate that toxins produced by symbionts can be transmitted along food chains, and that symbionts can have significant impacts in species other than their host.

Though in most systems in which symbionts negatively impact predators the microbes directly reduce predation through the production of either predation-deterring toxins *e.g.*[27] or growth inhibiting molecules *e.g.*[34], our findings highlight another mechanism by which symbionts can affect predation, namely by reducing the survival of the predator after predation. This may affect aphid fitness indirectly by reducing the local lady beetle larval population and therefore the number of emerging lady beetle adults. Aphids reproduce parthenogenically for most of their annual lifecycle and during the summer months live in patches of genetically identical individuals (or nearly genetically identical; see [35]) [36,37]. Additionally, female lady beetles generally lay small groups of eggs near developing aphid patches [38], and though larvae may disperse and search for food [39], empirical studies have found that lady beetle larvae mainly live in same-age groups, suggesting little dispersal for the majority of larvae [40]. Therefore, the reduction of local predator density could benefit groups of aphids that occupy the same plant or plants in the immediate vicinity. Such indirect effects could significantly impact aphid population dynamics and competition in natural systems [41,42].

Given the negative impact of aphid symbionts on lady beetle survival, it is surprising that lady beetles fed aphids with facultative symbionts as larvae were heavier at adult emergence than those fed aphids lacking facultative symbionts. We hypothesize several explanations for the correlation between increased adult size and reduced survival in our facultative symbiont-fed lady beetles. One possibility is that the presence of aphid facultative symbionts in the diet of lady beetle larvae has an adverse effect on lady beetle health, and therefore only larvae that are predisposed to be vigorous and large, either due to genetic or maternal effects, will survive to adulthood. Another possibility is that their mothers, which were also fed symbiont-harboring aphids, assessed the environment as less favorable and thus produced larger eggs that developed into larger adults, which have higher fitness under conditions of poorer resource availability [43,44]. In these cases, the decreased survival is correlated with, but not attributed to, larger size.

Another intriguing follow-up to this current study would be to look at the fecundity of the adults. The presence of symbionts in the diet of developing lady beetles may affect a female’s reserve of eggs in adulthood. This would be particularly true if size and reproductive investment represented a trade-off for larvae stressed by their symbiont harboring aphid diet. Alternatively, if fecundity is correlated with female weight in this species, as it is in other insects [45], then increased adult weight and therefore fecundity due to the consumption of aphids harboring symbionts may off-set any survival effects. Future studies examining fecundity and offspring survival would allow us to better assess the possible effects of aphid symbionts on lady beetle population growth.

In the case of feeding rate, we saw no difference in the rate at which lady beetles consumed aphids with and without facultative symbionts when not given a choice. If natural populations of lady beetles experience the negative impact of survival associated with feeding on aphids with facultative symbionts found in this experiment, then selection may be expected to shape lady beetle preferences towards feeding on aphids without facultative symbionts if given a choice. One of the focal symbionts here, *H. defensa*, alters the production of alarm pheromones by aphids [46], providing one mechanism by which lady beetles could discriminate between aphids with and without symbionts. Future work should assess if either larval or adult lady beetles exhibit symbiont-associated prey preferences or avoidance behaviors. Additionally, both males and females should be studied in order to determine if there are differences in predatory behaviors. If larger females are more fecund and aphid symbionts significantly affect adult lady beetle size, then females might benefit from selecting aphids with symbionts, despite their negative survival effects.

## Conclusions

Several symbionts associated with both plants and animals protect their hosts by deterring predation. This study highlights another mechanism by which symbionts can protect their hosts from predation. Though the predatory lady beetles were not deterred from eating aphids harboring facultative symbionts, by lowering beetle survival and consequently reducing the local population of lady beetle larvae, the risk of predation is reduced for nearby clone mates [47,48]. This indirect protection is yet another defensive role for the aphid facultative symbionts *H. defensa* and *S. symbiotica*, and highlights the impact of symbionts, not just on the fitness of their host species but throughout the food chain.

## Authors’ contributions

JK, NMG, AK and KC designed the experiments. JK, AK and KC carried out the experiments. JK and NMG analyzed the data. All authors wrote and approved the manuscript.
